# Age as a risk factor in vasculitis

**DOI:** 10.1007/s00281-022-00911-1

**Published:** 2022-02-09

**Authors:** Andrea D. Gloor, Gerald J. Berry, Jorg J. Goronzy, Cornelia M. Weyand

**Affiliations:** 1 Department of Medicine, Stanford University School of Medicine, Stanford, CA, USA; 2 Department of Pathology, Stanford University School of Medicine, Stanford, CA, USA; 3 Mayo Clinic Alix School of Medicine, College of Medicine and Science, Rochester, MN, USA

**Keywords:** Giant cell arteritis, Takayasu arteritis, Immunosenescence, mTOR, CD8^+^ Treg cells, NOTCH, Inflammaging

## Abstract

Two vasculitides, giant cell arteritis (GCA) and Takayasu arteritis (TAK), are recognized as autoimmune and autoinflammatory diseases that manifest exclusively within the aorta and its large branches. In both entities, the age of the affected host is a critical risk factor. TAK manifests during the 2nd–4th decade of life, occurring while the immune system is at its height of performance. GCA is a disease of older individuals, with infrequent cases during the 6th decade and peak incidence during the 8th decade of life. In both vasculitides, macrophages and T cells infiltrate into the adventitia and media of affected vessels, induce granulomatous inflammation, cause vessel wall destruction, and reprogram vascular cells to drive adventitial and neointimal expansion. In GCA, abnormal immunity originates in an aged immune system and evolves within the aged vascular microenvironment. One hallmark of the aging immune system is the preferential loss of CD8^+^ T cell function. Accordingly, in GCA but not in TAK, CD8^+^ effector T cells play a negligible role and anti-inflammatory CD8^+^ T regulatory cells are selectively impaired. Here, we review current evidence of how the process of immunosenescence impacts the risk for GCA and how fundamental differences in the age of the immune system translate into differences in the granulomatous immunopathology of TAK versus GCA.

## Introduction

The healthy immune system has multiple mechanisms in place to protect the heart and the large blood vessels from inappropriate inflammation, a mechanism often called immune privilege. Accordingly, autoimmune disease of the myocardium, the aorta, and the large elastic arteries is rare [1, 2]. Insufficiently controlled vascular inflammation is now recognized as a major disease component of atherosclerotic disease. Treatment trials have demonstrated that inflammatory cytokine IL-1 blockade is sufficient to protect at-risk patients from recurrent cardiovascular events [3] and the anti-inflammatory medication colchicine has been shown to be vasoprotective [4]. Also, recent evidence that clonal hematopoiesis is a strong risk factor for atherosclerotic disease emphasizes the pathogenic connection between malfunction of myeloid immune cells and inflammation in the atherosclerotic plaque [5-7]. Among inflammatory diseases affecting the aorta and its major branches, atherosclerotic disease is frequent while autoimmune vasculitides are rare. The risk for both disease categories is strictly age-dependent. Atherosclerotic disease and giant cell arteritis (GCA) occur in older individuals, whereas Takayasu arteritis (TAK) is a disease that affects primarily young individuals. Age is such an important risk factor that it has been integrated as a classification criterion for both GCA and TAK. The age range for TAK has been set as below 40 years of age; some patients may have onset of symptoms in their early forties. GCA is diagnosed exclusively in over 50-year-old individuals [8, 9]. How young age predisposes to TAK and old age influences susceptibility to GCA or atherosclerosis are not well understood.

Age as a disease risk factor is increasingly recognized as the older adult population expands, and age-related morbidities create an increasing burden of disease. The recent COVID-19 pandemic has exemplified host age as a major determinant of disease outcome [10, 11]. In 2019, the average life expectancy of a 60-year-old American was 83.1 years, meaning that nearly one-third of life is lived beyond age 60 [12]. While it is still unclear whether organ systems age in tandem, it is undebatable that aging of the immune system is intimately linked to aging-related morbidities, including the exponentially higher risk of older individuals to develop cardiovascular disease, cancer, and neurodegenerative disease and to succumb to infections [10, 13]. With increasing age, tissues undergo structural and functional alterations, and the innate and adaptive immune system deteriorates by immunosenescence [10, 14]. In addition to weakened immune defenses against infections and decreased response to vaccinations, the aging immune system tends to become more inflammatory and more autoreactive. The chronic low-grade inflammation associated with immune cell age is now recognized as “inflammaging.” The term captures the co-occurrence of aging with the increased propensity of inflammatory immune responses. In turn, inflammaging alters tissue structures and metabolic and immune functions [14-16]. Together, they increase the threat of a broad spectrum of diseases and organ failure [10, 17]. It is now understood that the immune aging process begins in the 3rd decade of life and accelerates after the age of 50 years [18-20]. As a rule, the adaptive immune system is immature during early life, matures during the 1st and 2nd decade of life, and remains highly functional over the next three decades. Innate immune responses, mediated by the myeloid cells of the hematopoietic system, are well balanced during early and middle life but become unopposed in older individuals, rendering the host susceptible to uncontrolled tissue inflammation.

Here, we will review the advances in understanding the aging of the human immune system, how immunosenescence can cause immunopathology with an emphasis on age-related restructuring of the immune system in GCA and TAK.

## Aging of the immune system

The immune system protects the integrity of the host by recognizing and destroying infectious, neoplastic, and other injurious agents. Humans possess two major tightly interlinked immune subsystems, the innate and the adaptive systems. The innate system is responsible for swift immunity by utilizing inherited receptor structures and is subject to high cellular turnover. Most innate immune cells live only hours to weeks requiring constant replenishment. Any aging-related changes in hematopoiesis will affect the production and functionality of myeloid cells and impact innate immunity. The adaptive immune system consists of clonally distributed T cells and B cells equipped with high-precision antigen receptors. T and B cells undergo sophisticated selection processes to minimize autoantigen recognition by their somatically acquired receptors. The need for innate and adaptive immune cells to provide lifelong protection against neoplasms and infections while providing lasting support for tissue repair and regeneration places these constituent inflammatory cells under enormous demand and proliferative pressures. T cells and B cells are the carriers of lasting immune memory, linking their long-term survival to the immunocompetence of the host. Immune cells are highly mobile and need to adapt to varying tissue environments, adding to cellular stress and attrition.

Both the innate and adaptive immune systems change profoundly with progressive age due in part to the everchanging landscape of exposures and the changing dynamics in immune cell generation and replenishment [19]. The immune systems of a 25-year-old patient with TAK and a 75-year-old patient with GCA are fundamentally different, yet both produce autoimmune tissue inflammation with granulomatous infiltrates. Both diseases are treated with immunosuppressive medications that reshape the immune system and challenge the patient’s ability to generate protective immune responses [21].

### Dendritic cells and macrophages

Most dendritic cells (DC) and macrophages (Mϕ) are myeloid cells that support immunity through a broad range of functional capabilities. Due to high-density HLA molecules on their cell surface and a specialized three-dimensional structure, DC are believed to be uniquely equipped to prime naïve T cells and provide the link between innate and adaptive immunity. Mϕ present antigens to T cells, mostly memory T cells but also participate in phagocytosis, efferocytosis, tissue cleanup, and secretion of cytokines, chemokines, enzymes, and metabolites. DC and Mϕ change phenotypically and functionally with age [22, 23]. In some studies, senescent DC release lower amounts of cytokines (type I interferons (IFNs) and tumor necrosis factor alpha (TNFα). In other studies, old DC like most myeloid cells that are replenished from old stem cells, acquire a more pro-inflammatory phenotype with high baseline cytokine production even in the absence of TLR triggering. Senescent DC are compromised in recruiting and priming naïve T cells, but remain competent in supporting pro-inflammatory T cells, specifically cytokine-dependent effector T cells [24]. DC are protectors of immune tolerance, and DC aging jeopardizes this function. Immature DCs continually sense self-antigens and contribute to self-tolerance by promoting Treg cell differentiation, whereas senescent DCs lose parts of their tolerogenicity and become immune-stimulating DCs [25].

The ability of somatic cells to enter the cell cycle and duplicate is limited. Replication-associated accumulation of damaged DNA, eroded telomeres, and aged mitochondria all result in cell death, or alternatively, cell cycle arrest and senescence [26, 27]. Data collected over the last decade have shown that senescent cells are far from non-functional but instead adapt and acquire new capabilities. Senescence-associated secretory phenotype (SASP) is characterized by the release of a variety of growth factors, cytokines, and pro-inflammatory mediators [28, 29]. In humans, Mϕ are end-differentiated myeloid cells and do not truly fulfill the criteria of senescence, e.g., entry into an irreversible cell cycle arrest. But, like other cell types, they adapt to a secretory program with age [13]. Accordingly, aged Mϕ are reported to respond to TLR stimulation with the secretion of high amounts of pro-inflammatory cytokines (TNFα, IL-6, and IL-1ß), generation of reactive oxygen species (ROS), and activation of the inflammasome [23]. In addition, data are emerging that the expression of co-stimulatory and co-inhibitory molecules on Mϕ is altered and tends to become dysregulated with age [30, 31]. The term “macrophaging” reflects the concept of Mϕ as a key driver for the subtle, chronic, subclinical inflammatory state of older individuals [15, 32], although the data is conflicting. Some studies of Mϕ from aged rodents have produced opposite results, with decreased production of pro-inflammatory cytokines and ROS following stimulation with LPS and IFNγ [33-35]. Some investigators have considered a potential role of chronic receptor stimulation resulting in Mϕ aging. This could be particularly relevant for damage-associated molecular patterns (DAMPs), endogenous nuclear, or cytosolic proteins released by damaged or dying cells. DAMPs are sensed by Mϕ, providing important information about the tissue microenvironment, and trigger a shift to a pro-inflammatory response designed to initiate tissue repair. As the host ages, exposure to infectious pathogens accumulates, forcing the hematopoietic system to keep up with the production of myeloid cells. Chronic exposure to DAMPs with aging could reprogram Mϕ, deviating them to become harmful instead of beneficial [36]. Exposure of hematopoietic cells to a variety of inflammatory and metabolic conditions triggers a program of trained immunity [37, 38]. Training, acquired in the bone marrow environment, enables Mϕ to retain long-term imprinting of microbial encounters. Epigenetic profiles, transcription factors, and micro-RNA networks have all been implicated in this process of reeducation of Mϕ [39].

A dominant mechanism through which aging can reshape innate immunity is clonal hematopoiesis [40]. Somatic mutations that accumulate with progressive age may provide a fitness advantage for some cells, e.g., mutated hematopoietic stem cells producing offspring with the competitive advantage will give rise to clonal populations, also known as clonal hematopoiesis of indeterminate potential (CHIP). Stem cell mutations ultimately lead to mutated immune effector cells, mostly monocytes and granulocytes. Studies have shown that CHIP is associated with an increased risk of atherosclerotic cardiovascular disease [41]. An excellent example of how aging-associated mutations in hematopoietic progenitor cells can lead to uncontrolled tissue inflammation is VEXAS syndrome. Somatic mutation in the UBA1 gene of hematopoietic progenitor cells causes a chronic, progressive autoinflammatory disease in older men with dermatologic, vascular, and hematologic manifestations [42, 43]. To date, there is no evidence of a parallel disorder involving clonal granulocyte and monocyte populations which are relevant in the age group susceptible to GCA.

### T cells and B cells

Immune memory, including recalling previous encounters to pathogens and vaccines, can last a lifetime. Essentially, all autoimmune diseases depend on the induction of T cell and B cell memory, which creates a particular therapeutic hurdle, i.e., the need to eliminate immune memory before true remission can be reached. The reliance of autoimmunity on T and B cells identifies the immune aging process as a critical component of disease risk. As a rule, B cells require help from T cells to develop into antibody-producing effector cells, rendering them highly susceptible to aging-imposed deficiencies in the T cell compartment. B cells undergo age-related changes in subset distribution and function. Older individuals expand a highly differentiated pro-inflammatory B cell phenotype with reduced ability to mount efficient antibody responses [16]. Mechanisms of immunosenescence are much better understood for T cells.

The adult human body possesses about 4 × 10^11^ T cells [43]. The immune system has mechanisms to maintain T cell numbers, with the exception of the last decade of life, when circulating T cell numbers decline noticeably and very old individuals develop frank lymphopenia. Based on studies in peripheral blood, the T cell receptor repertoire in healthy humans contains 10^7^ to 10^8^ different clonotypes, with clear age-imposed compression of diversity [44, 45]. In patients with rheumatoid arthritis (RA), the TCR repertoire is tenfold smaller, supporting the concept that RA is associated with premature immune aging [46].

To achieve preservation of the T cell pool and to secure protective immune memory, the host must balance T cell loss and regeneration. Given the constant demand for T cells to fight infections and malignant cells, aging imposes a lymphoreplete condition. Humans deal with this problem by producing new T cells in the thymus during the first and second decade of life and then switch to T cell replenishment by the homeostatic proliferation of circulating post-thymic T cells [47, 48]. By the third decade of life, the involuted thymus contributes only a minor proportion of the T cells produced daily. Instead, T cell loss imposes proliferative pressure on available T cells. Human T cells express telomerase and can, to some extent, repair telomeres; however, as somatic cells, T cells can only undergo a limited number of divisions [49]. This is the major hurdle for T cell replenishment in older adults. In contrast, mice replenish their T cell compartment exclusively by thymic output [50], sharply limiting their use in studies of immune aging.

T cells are among the body’s longest-lived cells and can survive from childhood to advanced age. Nucleotide sequencing studies in identical twins have identified shared T cell clonotypes that persist for > 50 years and represent the progeny of T cells generated during fetal development [51]. While some long-lived T cells may actually persist as quiescent cells, many will be constantly regenerated by proliferation from the original clone. The coordinated action of homeostatic T cell proliferation and lifelong antigenic challenge leads to skewing of the T cell repertoire toward differentiated effector and memory T cells, considered a hallmark of immune aging [20, 22]. Some viral pathogens, specifically members of the Herpesviridae family, cause chronic infection and co-exist with the host. Accordingly, cytomegalovirus (CMV), Epstein-Barr virus (EBV), and varicella-zoster virus (VZV) are likely modulators of the adaptive immune system in the aging adult [52, 53].

Continuous proliferative pressure and persistent antigenic stimulation will eventually shrink the naïve T cell pool and increase the number of end-differentiated CD4^+^ and CD8^+^ cells, so-called TEMRA cells, considered late-stage memory T cells [20, 54]. Another outcome of immune stress is T cell senescence or exhaustion, major determinants in the reduced capacity to eliminate cancer cells in older individuals [55-57]. Accumulation of end-differentiated CD4^+^ T cells is a hallmark of the immune system in patients with atherosclerotic disease, where these “old” T cells function as tissue-residing IFN-gamma producers [58-60] and mediate cytotoxicity against endothelial cells [61].

The propensity of the aged immune system to tolerate inflammatory activity may reflect the diminished function of regulatory T (Treg) cells. Tregs function as critical regulators of tolerance and autoimmunity by controlling the intensity and duration of innate and adaptive immune responses. Like all T cells, Tregs are subject to the aging process and their repertoire and function decline with progressive age [62]. Although the percentages and absolute numbers of CD4^+^ and CD8^+^ Treg cells are higher in older individuals, inducible CD4^+^ and CD8^+^ Tregs are reduced, and their functional activity is impaired [62]. Aged CD4^+^ Tregs are less capable of controlling syngeneic CD4^+^ T cells but still suppress allogeneic CD4^+^ T cells. Thus, senescent CD4^+^ Tregs may fail to protect against autoimmunity [63]. Evidence has emerged that the less studied CD8^+^ Tregs hold an important position in protecting the host from autoimmunity [64]. Like CD4^+^ Treg cells, aged CD8^+^ Tregs show a reduced ability to suppress proliferation and cytokine production of CD4^+^ effector T cells. A population of exosome-producing CD8^+^ Treg cells appears particularly susceptible to aging-induced loss-of-function [65]. These cells derive from naïve CD8^+^ T cells, and of all T cell populations, naïve CD8^+^ T cells are the most susceptible to the aging process [66].

**Fig. 1:**
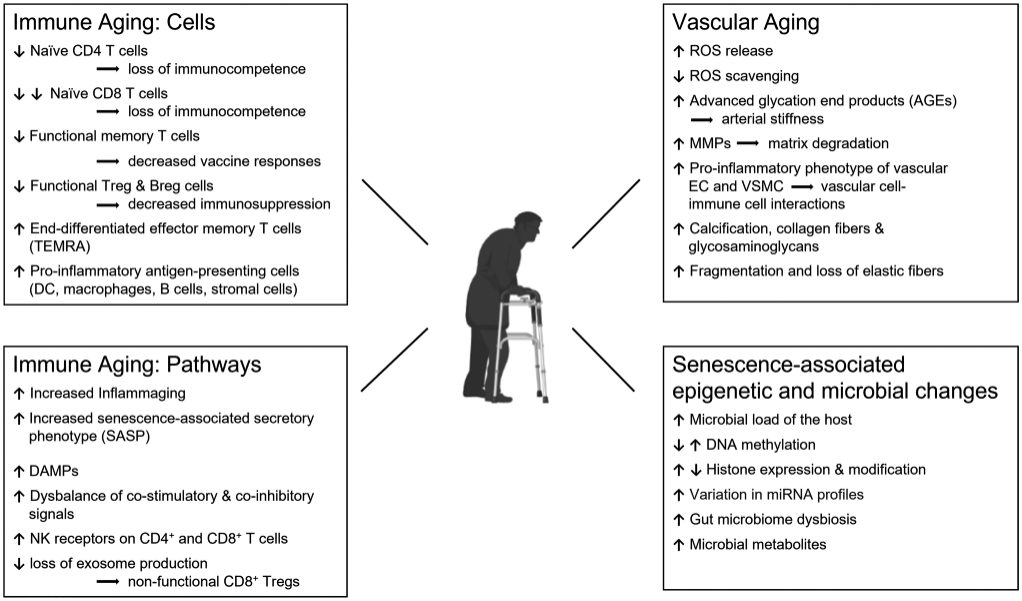
The interface of aging and vascular inflammation. The cellular and molecular pathophysiology of organismal aging is becoming increasingly understood, and it is now recognized that the aging process is a major disease risk factor. Most aging-associated morbidities are closely connected to the failure of the immune system, including the host’s inability to fight infections and cancers and to repair and maintain intact tissues. The aging immune system loses competence and shifts toward unopposed inflammatory activity, a process called “inflammaging.” The risk of older adults to develop autoimmune vasculitis relates to progressive immune aging, structural changes in the tissue environment of the vessel wall, alterations in genetic and epigenetic stability, and aging-induced adaptations of the host microbiome

The last decade has produced major advances in identifying mechanisms of lymphocyte differentiation into memory T and B cells and clarified where and how memory cells persist [67]. Tissue-resident memory T cells (Trm) survive and act locally in barrier tissues and respond promptly to antigen reexposure, e.g., reinfection. They depend on specialized tissue niches to support their fitness, function, and plasticity. How tissue aging impacts Trm is not well understood, but emerging data support a role for tissue-deposited memory T cells in inducing immunopathology in autoimmune inflammation [68]. Figure 1 highlights the major shifts in the cellular composition of the immune system over a lifetime and summarizes current knowledge about pathways that are relevant in immune aging.

## Age-related changes in blood vessels

**Fig. 2:**
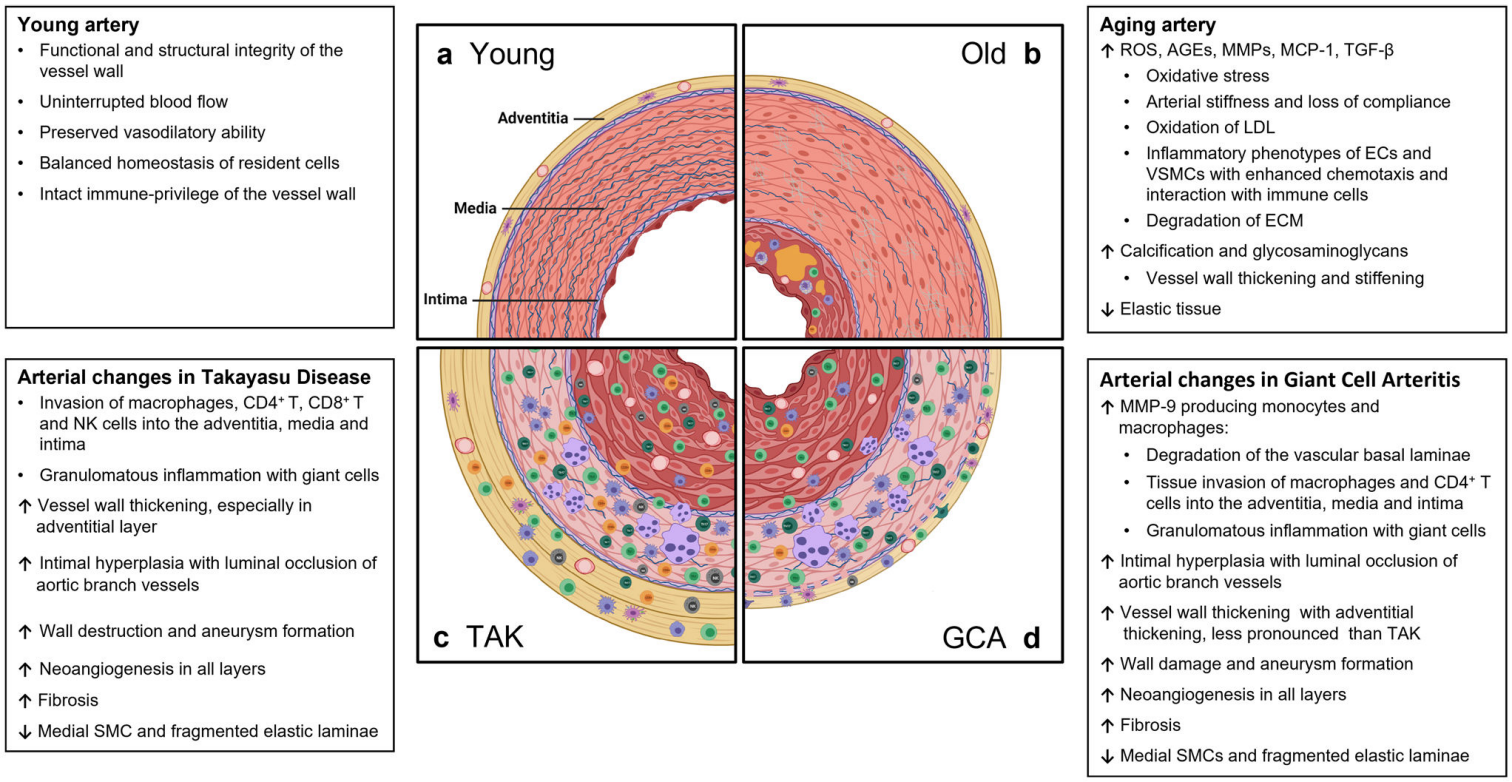
Vascular microenvironments in young, old, and inflamed arteries. Medium and large arteries must withstand lifelong mechanical stress, requiring maintenance of vasoconstrictor/vasodilator capacity and preservation of anticoagulant and anti-inflammatory surfaces. The vessel wall is an immunoprivileged tissue niche protected from the invasion of inflammatory cells. In the medium/large-vessel vasculitides, Takayasu arteritis and Giant cell arteritis, the immune privilege of the vessel wall is broken, and macrophages and T cells invade into the adventitia and media to form granulomatous infiltrates. The vessel wall responds with a maladaptive response-to-injury, resulting in thickening of the adventitia, intramural neoangiogenesis, and intimal hyperplasia. Clinically relevant outcomes are arterial stiffening, aortic aneurysm formation, and ischemia-inducing luminal stenosis of aortic branch vessels. Cellular infiltrates are shaped by the host immune system, containing CD4^+^ and CD8^+^ T cells in the young TAK patients and predominantly CD4^+^ effector T cells in the old GCA patient

The vascular system is a life-sustaining organ composed of networks of arteries, arterioles, and capillaries transporting blood containing oxygen, micro- and macronutrients, metabolic end-products, and immune and progenitor cells [69]. The vascular inner intimal layer directly interfaces with a wide range of external and internal stimuli. In addition, medium and large arteries have a constituent vascular support system, the vasa vasorum. Localized within the adventitia, vasa vasora provide access for oxygen and nutrients to the vessel wall layers and act as the interface between the target tissue and the infiltrating immune cells in large-vessel vasculitis [70]. Vascular disease is the leading cause of aging-associated morbidity and mortality in the USA. By far, the most frequent disease process manifesting in aging blood vessels is atherosclerosis, but little is known about the precise steps of vascular failure induced by progressive age [71, 72]. Aging entails various detrimental effects on extracellular and cellular components in vascular tissues. Much emphasis has been put on the potential injurious impact of reactive oxygen species, but therapeutic attempts to ameliorate cardiovascular disease with antioxidant therapies have proven largely unsuccessful [73]. With age, the disturbed balance between the production and neutralization of ROS leads to oxidative damage to DNA and cellular structures and changes in intracellular signaling [74]. Advanced glycation end-products (AGE), which are markedly increased with age, are formed by a nonenzymatic reaction of proteins, lipids, or nucleic acids with sugar molecules. AGEs can produce arterial stiffness and vessel wall damage by inducing crosslinking of extracellular matrix proteins, oxidation of low-density lipoprotein (LDL), and promoting inflammatory pathways in endothelial cells (EC) [75]. Arterial wall remodeling associated with aging also involves matrix metalloproteinases (MMPs), which degrade the extracellular matrix (ECM) and activate pro-inflammatory molecules such as monocyte chemoattractant protein-1 (MCP-1) and transforming growth factor-beta 1 (TGF-β1) [76, 77]. Senescent EC and vascular smooth muscle cells (VSMC) adopt a pro-inflammatory phenotype and show altered interaction with immune cells (e.g., enhanced chemotaxis and expression of adhesion molecules such as VCAM1 and ICAM1) [78]. Other structural changes including fragmentation and loss of elastic fibers, mural calcification, increase of collagen fibers and accumulations of glycosaminoglycans in the medial layer, and expansion of smooth muscle cells and extracellular matrix in the intimal layer produce vascular thickening, diminished compliance, and stiffening (Figs. 1 and 2) [79]. How these structural changes participate in the molecular processes that disrupt the immune privilege inherent to the walls of the large elastic arteries is currently unknown.

### Age-dependent immunopathology in GCA and TAK

GCA is a granulomatous arteritis affecting the 2nd–5th branches of the aorta. Inflammatory lesions are centered on the aortic and arterial wall and are composed primarily of highly activated macrophages and T cells eliciting a maladaptive response-to-injury mechanism. The disease is exclusively diagnosed in individuals over 50 years of age. The average age in cohorts of GCA patients lies in the middle of the 8th decade of life, with a remaining life expectancy of less than 10 years. The disease has an extravascular component, presenting with an intense acute phase response, highly elevated hepatic acute phase proteins, myalgias, and constitutional symptoms such as painful stiffness of select muscular territories, described as polymyalgia rheumatica (PMR), fever, weight loss, and failure-to-thrive [80, 81].

**Fig. 3:**
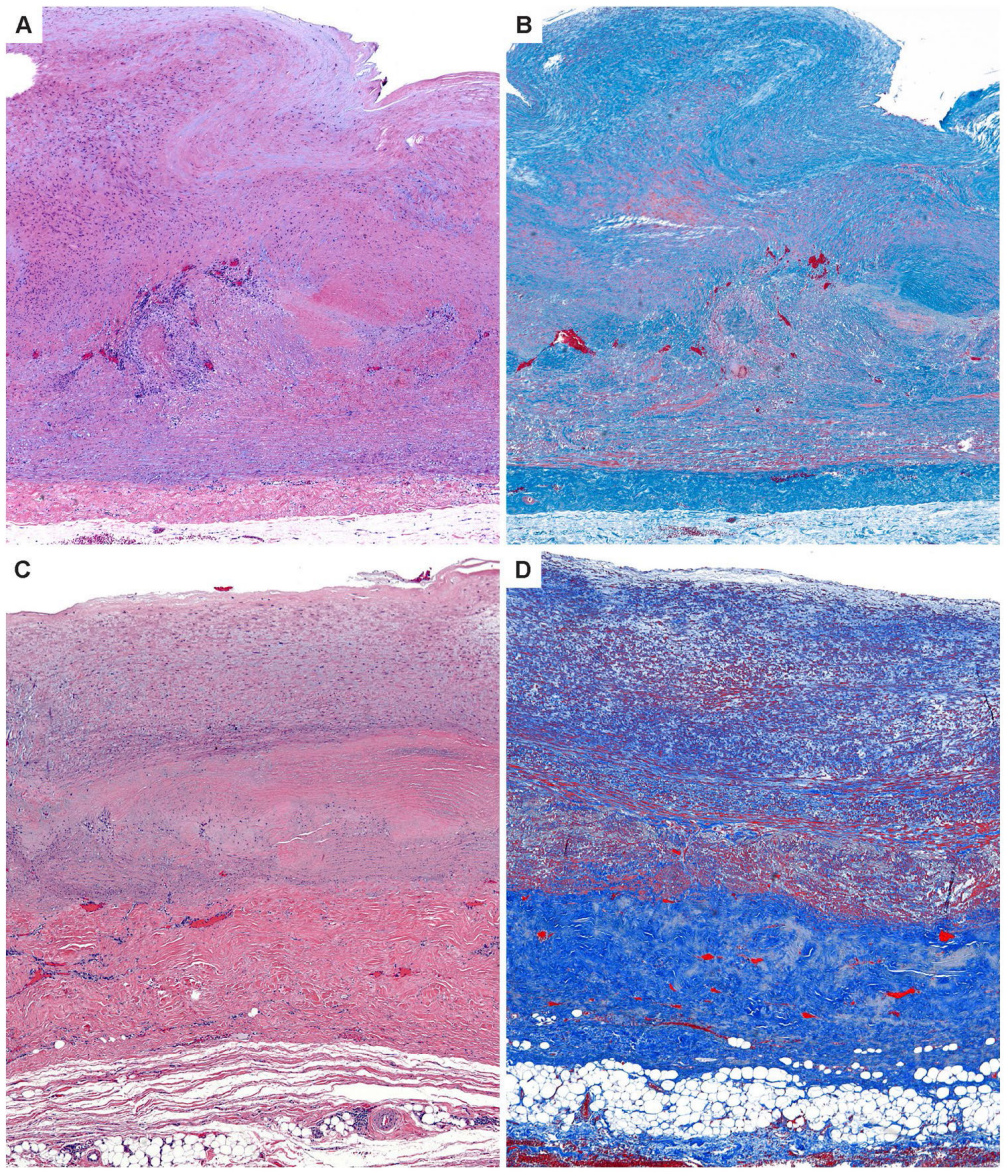
Histopathology of giant cell aortitis and Takayasu aortitis. **A**, **B**. Giant cell aortitis in a 70-year-old woman with an ascending aortic aneurysm is characterized by pan-arterial injury with intimal hyperplasia, inflammation and zones of medial necrosis, and mild fibrous thickening of the adventitial layer (H&E and trichrome stains). **C**, **D**. Takayasu aortitis in a young woman presenting with a murmur and ascending aortic aneurysm. There is conspicuous mural thickening especially of the adventitial layer along with granulomatous inflammation of the medial layer (H&E and trichrome stains)

**Fig. 4:**
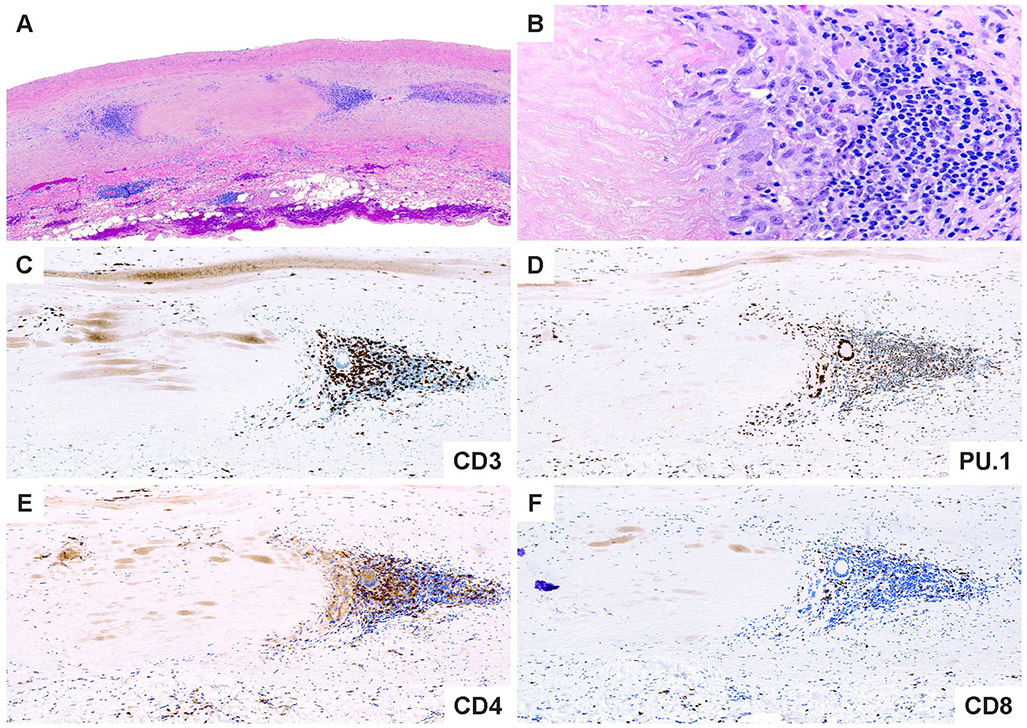
Immunopathology of giant cell arteritis. **A**. Low power magnification of GCA characterized by numerous foci of medial inflammation and destruction along with intimal and adventitial inflammation and thickening. **B**. High power magnification showing an admixture of macrophages including multinucleated cells and lymphocytes surrounding the zone of medial necrosis. **C**. CD3 ^+^ T cells comprise the majority of cells within the medial layer. **D**. PU.1 ^+^ macrophages including the multinucleated giant cells. **E**. The T cell infiltrates are composed primarily of CD4 ^+^ T cells. **F**. Only scattered CD8 ^+^ T cells are found in the intimal inflammatory focus

**Fig. 5:**
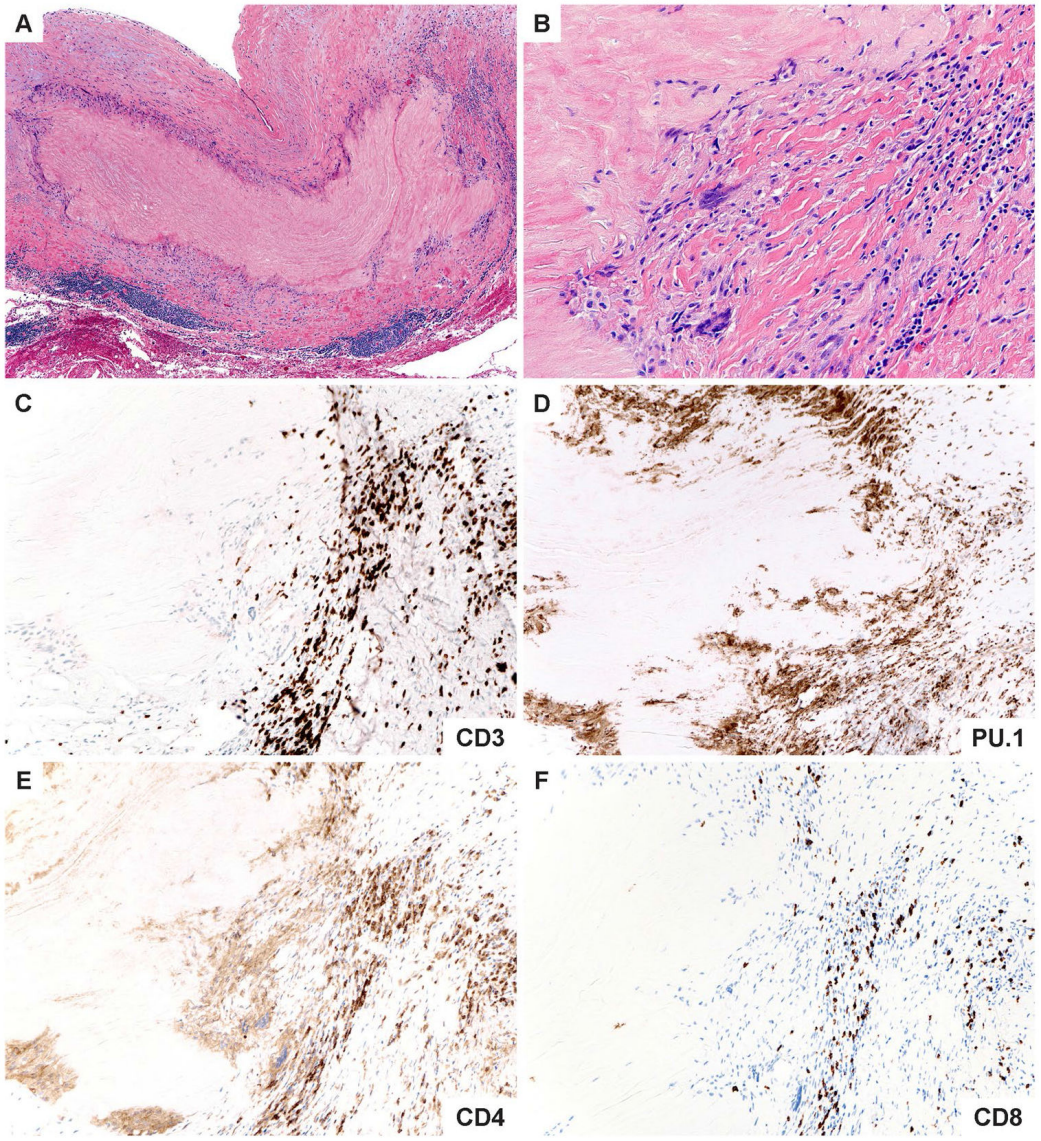
Immunopathology of Takayasu aortitis. **A**. Low power magnification of TAK aortitis. The intimal and adventitial layers are expanded by fibroinflammatory tissues. The medial layer shows discrete zones of necrosis surrounded by variable inflammatory collars. **B.** High power magnification showing a cuff of mononuclear cells including multinucleated giant cells and lymphocytes around the necrotic medial layer. **C.** Abundant CD3 ^+^ T cells. **D.** Numerous PU.1 staining macrophages. **E.** CD4 ^+^ T cells within the medial inflammatory cluster. **F.** CD8 ^+^ T cells are disproportionately increased compared to GCA

While the vascular territory and the clinical manifestations are largely overlapping, GCA and TAK affect two non-overlapping patient populations. Like GCA, TAK is a granulomatous arteritis of the aorta and its primary branches [80, 82]. However, there are key differences in the composition of the inflammatory infiltrates as well as in the histological appearance of the inflamed vessel wall [21], suggesting similarities and differences in the pathomechanisms underlying both diseases. In the active phase, TAK is characterized by granulomatous aortitis with distinctive thickening of the medial and adventitial layers compared to GCA, resulting in the imaging changes in the ascending aorta (Figs. 2 and 3). Typically, the granulomatous infiltrates of TAK contain increased proportions of CD8^+^ T cells and natural killer cells, suggesting a role for cell-mediated toxicity (Figs. 3 and 4) [21]. This is not unexpected, given that the granulomatous lesions of TAK are formed by a younger immune system, as opposed to the granulomatous infiltrates of GCA-affected arteries where CD8^+^ T cells are at low abundance due to aging-imposed restructuring of the immune system (Fig. 5) [83]. TAK can affect teenagers with a peak incidence in the third decade, a period wherein the immune system is still supplied by high thymic output. A diagnosis of TAK in individuals older than 40 years should only be considered if there is evidence for disease onset prior to 40 years of age. Currently, there are no data indicating prematurity of the immune senescence process in either TAK or GCA patients, in contrast to rheumatoid arthritis [84]. Age is also a recognized risk factor in Kawasaki’s disease (KD), a febrile illness that primarily affects children younger than 5 years of age and can lead to vasculitic destruction of coronary arteries. Seasonality and community-wide outbreaks in KD have supported the concept that this vasculitis is caused by a maladaptive immune response against an infectious pathogen. Thus, studies in the vasculitides provide an opportunity to examine the impact of chronic inflammatory disease on the immune aging process itself and permit comparisons of pathogenic capabilities of the old versus the young immune system.

### Pathogenic dendritic cells and macrophages in large-vessel vasculitis

Myeloid cells are subject to aging, and comparing myeloid cell populations in GCA and TAK could be informative in identifying specific characteristics of macrophages and dendritic cells in granulomatous inflammation. Dendritic cells that accumulate in the inflamed vascular wall are believed to be of mixed origin, derived partially from the endogenous population of vascular DC within tissues and partly from tissue-infiltrating DC originating in the bone marrow [85]. In GCA, they are strategically localized between the adventitia and the media, in close proximity to the vasa vasorum through which infiltrating immune cells reach the target tissue. DC are also part of the cellular infiltrate in atherosclerotic plaque, where they secure in situ recognition of antigens [86]. DC are professional antigen-presenting cells required for the priming of naive T cells. The activation status, and thus their ability to take up and process antigen, is ultimately determined by the recognition of pathogen-derived molecular patterns (PAMP) and danger-associated molecular patterns (DAMP), a process involving Toll-like receptor (TLR) molecules. TLR expression profiles of vascular DC have been obtained for different types of arteries and demonstrate a close correlation between vascular territory and DC function [87]. This territorialism may be critically important in determining the tissue tropism in GCA. Similar studies are currently unavailable in TAK. The best evidence that antigen recognition occurs in the inflamed vessel wall stems from shared T cell clonotypes in the right and left temporal artery biopsies from GCA patients [86]. Again, information on antigen recognition events in the granulomatous infiltrates of TAK is still needed.

Besides the presentation of HLA-embedded antigenic peptides on their surface, DC guide the antigen-presenting process by providing both positive (co-stimulatory) and negative (co-inhibitory) signals. In GCA, the tissue-resident DC are strongly positive for CD80 and CD86 [88] and the disease process is shaped by CD28–CD80/CD86 interactions [89]. CD28 signaling has been implicated in regulating the metabolic profile of vasculitic T cells, favoring glycolytic activity but also mitochondrial respiration [89]. Tissue-residing T cells in GCA are hypermetabolic and depend on an ample supply of glucose and alternative energy carriers [89]. Implicating dendritic cells in setting the threshold for energy consumption of pathogenic T cells builds an immediate bridge to aging-imposed mechanisms, as the metabolic programming of T cells is an age-dependent phenomenon [19].

A key functional abnormality of DC in GCA is the low expression of the co-inhibitor molecule PD-L1 [90]. In GCA-positive temporal artery biopsies, vessel wall-residing DC have barely detectable PD-L1. The defect appears to be systemic; DC generated from bone marrow-derived precursors share the PD-L1 low phenotype [90]. PD-L1 on antigen-presenting cells cross-links PD-1 on T cells to transmit a negative signal. PD-1 is critical in shaping an array of T cell functions, including the induction of T cell anergy, T cell exhaustion, T cell apoptosis, as well as the survival of Treg cells [90, 91]. The importance of the PD-1/PDL-1 signaling pathway in determining the strength and duration of T cell responses has further elucidated with the therapeutic application of immune checkpoint inhibitors in cancer. Antibodies blocking access to either PD-1 or PD-L1 disrupt excessive signaling in this pathway and unleash T-cell immunity against malignant cells. Notably, cancer patients treated with checkpoint inhibitors can develop a spectrum of autoimmune manifestations, including large-vessel vasculitis [92, 93]. In GCA, PD-L1^low^ vascular DC fail to block the differentiation of effector T cells in the vasculitic lesions, leading to the accumulation of interferon-gamma, IL-17, and IL-21-producing T cells [90]. There is currently no data on the functionality of the PD-1/PD-L1 pathway in TAK. However, the shared deficiency of PD-L1-dependent signaling in cancer and GCA occurs in the setting of advanced age, possibly identifying the failure of this important co-inhibitory pathway as an aging-related pathology.

Macrophages (Mϕ) are a core constituent of the granulomatous lesions in GCA (Fig. 4) and TAK (Fig. 5) and can transition into multinucleated giant cells [80]. As end-differentiated myeloid cells, Mϕ are highly susceptible to bone marrow aging. Mϕ encountered in inflammatory tissue lesions are heterogeneous, characterized by distinct origins, fate decisions, and differentiation pathways [94, 95]. A comprehensive analysis of the Mϕ heterogeneity in the vasculitic infiltrates is lacking, but currently, the majority are presumed to originate from the bone marrow. Monocyte-derived Mϕ from GCA patients demonstrate abnormalities in two domains, the production of metalloproteinases, specifically, MMP-2 and MMP-9 and the propensity to produce chemokines [96, 97]. The ability of GCA Mϕ to produce active MMP-9 has been mechanistically linked to tissue invasion in in-vitro and in-vivo studies. Importantly, T cells could not penetrate basal membranes unless supported by MMP-9^+^ Mϕ [96]. High expression of MMP-9 persists in multinucleated giant cells, suggesting direct involvement in the digestion of vessel wall structures [96]. Antibodies capable of specifically inhibiting the activity of MMP-9 prevented invasion of T cells into the vessel wall, and more importantly, they inhibited the wall remodeling process, including neovascularization and intimal hyperplasia [96]. These data support the concept that MMP production is not simply an epiphenomenon of macrophage activation but is mechanistically linked to the disease process. Whether dysregulation of MMPs also contributes to the pathophysiology of TAK is still controversial. Studies found increased levels of several MMPs (e.g., MMP-2, −3, −9) in the blood of TAK patients; other studies did not support these findings [98]. MMPs have been implicated in the process of vascular aging by directly contributing to wall fibrosis and stiffening [99, 100]. A propensity of GCA and possible TAK monocytes and macrophages to release excessive MMPs may be relevant for the loss of tissue tolerance and, in a feed-forward loop, lead to accelerated aging of the affected vessels.

Despite the hyperactivated state of macrophages participating in the formation of mural granulomatous inflammation, metabolic studies have found that GCA macrophages are very similar to age-matched controls [97]. Notably, comparative studies of macrophages in GCA and in coronary artery disease (CAD) have shown striking metabolic differences with high-intensity glycolytic activity and mitochondrial respiration selectively in nonvasculitic CAD patients [97]. While GCA and CAD are diseases of older individuals and dependent on Mϕ for their respective pathophysiology, risk factors and disease mechanisms appear to be disease-specific and not a consequence of immune and vascular aging. The pro-inflammatory state of CAD Mϕ has been linked to ROS-induced metabolic reprogramming [101]. Mitochondrial hyperreactivity leads to oxidative modification and dimerization of the glycolytic enzyme PKM2, which then translocates into the nucleus, where it activates STAT3 and promotes IL-1β and IL-6 production. High production of ROS and mitochondrial stress has also been described for Mϕ that are part of the GCA infiltrates [102]. High mitochondrial ROS release has been considered a hallmark of aging [103]. Applying that paradigm, CAD and GCA Mϕ are stressed and aged.

### Aberrant mTORC1 signaling in aging and in large-vessel vasculitis

**Fig. 6:**
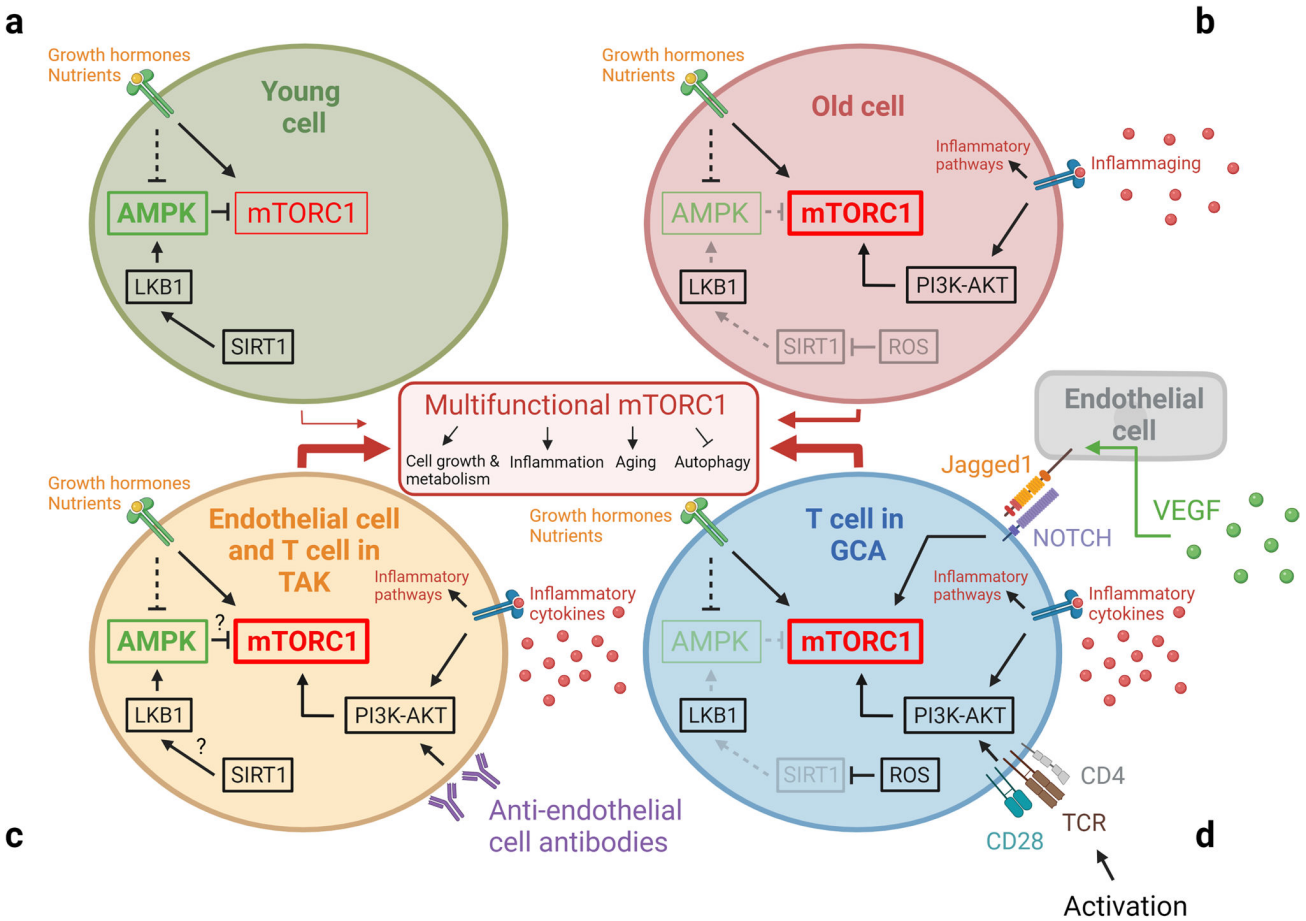
Aberrant mTORC1 activation on aging and in vasculitis. The mammalian target of rapamycin complex 1 (mTORC1) is the cell’s major nutrient/energy/redox sensor and coordinates protein synthesis with the cell’s bioenergetics. AMP-activated protein kinase (AMPK) activates catabolic and suppresses anabolic metabolism to secure energy balance. Signaling in the two pathways is closely inter-connected and inflammatory effector functions directly dependent on mTORC1 and AMPK activation. Increased activity of mTORC1 and diminished activity of AMPK is a hallmark of cellular aging, and the two kinases have become therapeutic targets to extend lifespan. Aberrant mTORC1 activation is now recognized as a typical feature of T cells in autoimmune vasculitis, where effector differentiation is sustained by signaling of the mTOR network. mTORC1 may also perpetuate the activation status of disease-relevant stromal cell populations

The mechanistic target of rapamycin (mTOR) is an evolutionarily conserved signaling hub that senses and integrates intracellular energy reserves and growth factor signals to align cell growth, proliferation, and death [104, 105]. The mTOR network measures and responds to the availability of intracellular nutrients and directs autophagy, mitochondrial function, and protein synthesis. mTOR complex 1 (mTORC1) and AMPK are now recognized as the two central metabolic sensors, meeting on the surface of the lysosome to determine cellular longevity and youthfulness versus senescence [106, 107]. Pharmacologic inhibition of mTORC1 is currently being explored as a therapeutic option to extend lifespan [108]. Increased activity of mTORC1 is a distinguishing feature of CD4^+^ T cells in both GCA and TAK (Fig. 6) [109], suggesting that these important regulators of granuloma formation have features of senescent T cells. The regulatory steps determining subcellular distribution and activity of the mTORC1 are complex. A variety of age-related as well as age-independent factors and signaling pathways influence mTORC1 activity [104]. Important mTORC1 regulators are the sirtuins (SIRTs). SIRTs are nicotinamide adenine dinucleotide (NAD^+^)-dependent deacetylases that have been implicated in aging and longevity [110]. SIRT1 inhibits mTORC1 by stimulating AMPK via LKB1. Lower NAD^+^ levels cause decreased SIRT1 activity in older individuals. PBMC from GCA patients shows lower SIRT1 activity when compared with age-matched healthy controls, possibly due to increased intracellular ROS levels [111]. Data on SIRT1 in TAK patients are not yet available. The Jagged1-NOTCH1 as well as the CD28-PI3KAKT pathway independently contribute to the regulation of mTORC1 activity, and both were found to be hyperactive in GCA T cells [89, 112, 113]. T cell specification and commitment depend on NOTCH signaling, and in post-thymic T cells, NOTCH signaling transmits a co-stimulatory signal. NOTCH has a role in a variety of other cell types, including macrophages, DCs, ECs, and VSMCs [114, 115]. Besides its essential role in cardiovascular development, the NOTCH signaling pathway has been implicated in multiple age-related processes and disease states. Notch receptors and ligands are considered to be responsive to inflammatory signaling and act as amplifiers of tissue inflammation by facilitating the crosstalk between immune cells (T cells, Mϕ) and vascular cells (EC, VSMC). Additionally, NOTCH1 appears to have a critical role in aortic aneurysm formation by enhancing Mϕ infiltration into the vascular tissue and increased MMP production [113, 116, 117]. In GCA patients, elevated VEGF levels induce the NOTCH ligand Jagged1 on microvascular endothelial cells, turning vasa vasora into communication partners for NOTCH1^+^ T cells [113, 118]. After interacting with Jagged1^+^ EC, NOTCH1^+^ T cells activate mTORC1 and turn into cytokine-producing effector cells, making a commitment to the Th1 or the T17 lineage.

Preferential differentiation of CD4^+^ T cells into Th1 and Th17 effector cells is a consistent feature in TAK patients, where it has been connected to aberrant mTORC1 activity. Hadjadj et al. have reported that the mTOR network is equally activated in EC from TAK patients, an abnormality not seen in GCA [119]. mTORC1 hyperactivity is believed to promote EC expansion, biasing T cell differentiation toward Th1 and Th17 polarization and impairing Treg cell function in both TAK and GCA. Taken together, mTORC1 hyperactivity is a consistent finding in T cells from TAK and GCA patients, connecting immune dysregulation with a signaling pathway typically seen in senescent cells. While part of this pathology may reflect immune aging in GCA, alternative upstream regulators must be at work in TAK. Anti-endothelial antibodies that are seen in TAK but not in GCA patients may function as EC activators and sustain the aberrant mTOR activation [120].

In GCA CD4^+^ T cells, aberrant mTORC1 activity can also be a consequence of disproportionate co-stimulation through the CD28-PI3K-AKT pathway [90]. While the surface expression of CD28 is similar in GCA and age-matched controls, the sensitivity to an anti-CD28 blocking antibody is much higher in the patients’ T cells [90]. CD28-dependent signaling shifted GCA T cells into a hypermetabolic state, enhancing survival, proliferative activity, effector cytokine release, and T-cell-induced macrophage activation [90]. Hyperactivity in the PI3K/AKT pathway has been described in neurodegenerative disorders, such as Alzheimer’s and Parkinson’s disease, two conditions strongly associated with aging [121]. Conversely, it could be argued that chronic activation of the NOTCH1 and PI3K/AKT pathway, both targeting mTORC1 as a downstream target, may simply reflect the chronicity of the disease process, leading to T cell exhaustion and an aging phenotype.

## Shared pathways in vasculitis and immune aging

Based on chronological age, TAK is a disease of the young and GCA is a disease of the old. In both vasculitides, age outranks other risk factors by far. Nevertheless, no formal analyses have been conducted assessing the phenotypic age of patients affected by TAK or GCA. TAK occurs early in life, making it highly unlikely that immunosenescence has a relevant contribution to disease risk. There are no data that could reliably assign TAK as a disease caused by premature aging. Rather, it is likely that the inflammatory process that persists over decades will have negative effects on immune health and impose a state of immune exhaustion. Furthermore, no studies have examined the impact of chronic vascular inflammation on the immunocompetence of TAK patients during old age. Models and concepts of the immunopathology of GCA cannot be separated from senescence-associated abnormalities in the immune system. GCA clearly manifests in individuals with an aging immune system, and aged Mϕ and aged T cells may excel in forming granulomatous lesions. Here, we will highlight processes that are well established in the field of immunosenescence that might transition individuals from at-risk to vasculitis patients. Table 1a and 1b summarize the adaptations in immune cell function in aging and in vasculitis.

### T cell senescence

Most convincing data derive from the comparative role of CD8^+^ T cells in GCA and TAK. CD8^+^ T cells and natural killer (NK) cells contribute substantially to the aortic wall lesions of TAK, while they are a minor population in the GCA-affected temporal artery and aorta [122, 123]. A cardinal feature of immune aging is the loss of CD8^+^ T cells. Within the same individual, CD8^+^ T cells age faster than CD4^+^ T cells and the accumulation of end-differentiated CD8^+^ CD45RA^+^ CCR7^−^ effector memory T cells (TEMRA) is a programmed event during the 2nd half of life [124]. Naïve CD8^+^ T cells exhibit a much greater decline in absolute and relative cell numbers and a higher expression of senescence markers with aging than CD4^+^ T cells [125]. Thus, the CD8^+^ T cell pool is much smaller in GCA patients than in TAK patients. Also, with progressive age, the CD4^+^ and CD8^+^ T cell compartments become enriched in CD28 negative, end-differentiated effector cells, so-called TEMRAs [20]. In parallel, CD4^+^ and CD8^+^ T cells evolve to a more NK cell-like phenotype with enhanced expression of NK receptors on their surface and increased cytotoxic capacity [20]. Enrichment of CD4^+^CD28^−^ T cells in the inflamed tissue provided the first clue that patients with rheumatoid arthritis have premature immune aging [126]. CD4^+^ CD28^−^ T cells are associated with unstable angina and acute coronary syndrome [59], accumulate within the atherosclerotic plaque [60] and function as cytotoxic T cells attacking endothelial cells [62, 127], highlighting the importance of TEMRAs in vascular inflammation. Conversely, NK cells lose their cytotoxic capacity while tending to expand with age (especially long-lived NK cells) [20, 128]. As expected, CD4^+^CD28^−^ T cells are increased in the peripheral blood and vascular lesions of GCA patients. These CD4^+^CD28^−^ T cells demonstrate upregulation of the NK receptor NKG2D [129], matching the senescent T cells previously described in rheumatoid arthritis [130] and in ANCA-associated vasculitis [130, 131]. Notably, in lupus, NKG2D^+^ CD4^+^ T cells appear to be immunosuppressive [132], but in GCA, NKG2D has been reported to enhance the pathogenicity of Th1 and Th17 cells and stimulate the release of pro-inflammatory cytokines such as IFNγ and Granulocyte–macrophage colony-stimulating factor (GM-CSF), which are both important pathogenic players [129, 133, 134].

### Treg cell aging

Treg cells are underestimated players in age-related diseases. The aging-induced decline of Treg numbers and function supports the general concept that the old immune system is pro-inflammatory. Failure of appropriately timed Treg-mediated immunosuppression has been considered relevant in the pathogenesis of GCA and TAK [81, 135]. It is difficult to draw functional conclusions from phenotypic studies, both in the blood and in the lesion, and model systems are needed that permit genetic and/or pharmacologic manipulations of Treg to properly assess their role in disease. CD8^+^ Tregs normally suppress surrounding CD4^+^ T effector cells by secreting NADPH oxidase 2-(NOX2) containing microvesicles, which inhibit their target cells by interfering with signaling pathways via ROS. The release of NOX2 and the resulting suppressive capacity is sharply reduced in CD8^+^ Tregs of older individuals and is even more pronounced in GCA patients [65, 66]. The aberrant NOTCH signaling pathway is responsible for this dysfunctional CD8^+^ Treg activity. NOTCH4, one of 4 different NOTCH receptors, mediates intracellular vesicle trafficking by regulating the expression of its target genes RAB5A, RAB7A, and RAB11A [136]. The Rab (Ras related in the brain) proteins are involved in the formation and recycling process of microvesicles and are crucial for intra- and extracellular communication [137]. Jin et al. have demonstrated an upregulation of NOTCH4 and altered RAB gene transcription in CD8^+^ Treg cells from GCA patients. The expression of RAB5A and RAB7A was enhanced, whereas RAB11A was suppressed, resulting in a failure of surface translocation and release of NOX2-containing microvesicles [136]. Blocking NOTCH4 signaling was sufficient to restore the immunosuppressive function of Tregs in vivo and to suppress vessel wall inflammation [136].

Forkhead box protein P3 (FoxP3), a transcription factor, functions as a regulatory protein in the development, polarization, and function of Treg cells. When alternatively spliced, FoxP3 generates four distinct isoforms determining the activity level and phenotype of Treg cells [138]. In GCA patients with active disease, peripheral CD4^+^ Treg cells preferentially express FoxP3Δ2 over the full-length isoform [139]. Tregs lacking exon 2 lose their suppressive function and even become pathogenic by producing IL-17 [140]. However, it is not known if this alteration has functional consequences in the disease process. Another mechanism that favors polarization toward Th17 cells over Treg cells is controlled by IL-6. IL-6, together with IL-21 and IL-23, blocks FoxP3 and stimulates RORγt, a transcription factor responsible for Th17 polarization [141, 142]. IL-6, a marker cytokine of older individuals [143, 144], is elevated in both GCA and TAK and could modulate Treg functions [81, 145].

There is limited data investigating the role of Treg cells in TAK, and their role in the pathogenesis of TAK differs from GCA [145]. Kong et al. have reported a reduction in circulating Tregs compared with controls, whereas Matsumoto et al. could not confirm this finding [145, 146]. Gao et al. found a decrease of circulating Th2-like Tregs, a dysfunctional Treg cell subset, in TAK patients [147]. The authors propose that the reduction in circulating Th2-like Treg cells might reflect a drift from the blood into the vascular wall, where they fail to control other immune cells and release pro-inflammatory cytokines.

### Age-associated cytokine production

An interesting and possibly age-related difference between GCA and TAK is effector cytokine production. In GCA, Th1 and Th17 are both encountered as effector cytokines in the inflamed vessel wall of GCA patients [148]. Lesional Th17 cells in GCA are responsive to corticosteroids, whereas Th1 cells persist despite prolonged treatment, suggesting that old IFN-γ-producing Th1 cells are resistant to immunosuppression. IFN-γ secretion may be one manifestation of T cell senescence. In contrast, Th1 cells are susceptible to corticosteroids, and Th17 cells are steroid-resistant in TAK [148, 149]. This may reflect a loss of plasticity in committed effector T cells in GCA versus TAK, compatible with immune aging as a disease risk factor.

### Age-dependent epigenetic regulation

An important aspect of the aging process is exposure to environmental stimuli which may leave long-lasting imprints in the immune system and in immuno-responsiveness. Epigenetic studies explore how environmental factors modify chromosomes without changing the underlying gene sequence, ultimately resulting in altered gene expression [150]. Epigenetic mechanisms regulate transcription factor accessibility to certain DNA regions and ultimately gene transcription. This is regulated by chemical modifications of DNA itself, such as methylation of cytosines, modification of histones, and by the interference of noncoding micro-RNA (miRNA) causing posttranscriptional gene silencing [151, 152]. Progressive age is associated with a variety of epigenetic changes, such as hyper- and hypomethylation of DNA, altered histone expression and modification, as well as dysregulation of miRNA expression [84, 153, 154]. DNA methylation is essential for the regulation of subset-specific T cell gene expression and generally declines with age, causing overexpression of certain genes. However, some tissue-specific CpG have also been found to be hypermethylated with age [84]. Epigenomic studies of human CD8^+^ T cells have yielded evidence for progressively declining chromatin accessibility at gene promoters with age, resulting in a decrease in NRF1 binding [155]. Old naïve CD8^+^ T cells failed to transcribe respiratory chain genes, causing a collapse in mitochondrial function, specifically in oxidative phosphorylation [155].

A genome-wide methylation array of GCA versus non-GCA temporal arteries revealed hypomethylation of signature T cell CpG related to TCR/CD28 signaling, proinflammatory cytokine production (e.g., IFNG, IL6, IL21, IL23R, IL17RA), and the calcineurin (CaN)/ nuclear factor of activated T cells (NFAT) pathway [156, 157]. These findings are in line with the accumulation of highly activated T cells in the vasculitic lesions. Alteration of miRNA expression contributes to aging, including endothelial dysfunction, vascular inflammation, and compromised angiogenesis. A single miRNA can control the expression of multiple genes by inducing mRNA degradation or inhibiting the translation of certain mRNA strands [158]. miR-21, miR-146a, miR-146b-5p, and miR-155 appear to be upregulated in actively inflamed GCA temporal artery biopsies compared to non-active GCA and healthy arteries [159]. However, miRNA target protein expression was indistinguishable among the groups, and miRNA expression patterns in peripheral PBMCs were similar in all three populations. In essence, DNA methylation and miRNA expression appear to be altered with age and in GCA patients; however, the impact of these alterations on GCA pathogenesis remains to be clarified.

Most genetic risk variants found in GCA and TAK are clustered in noncoding DNA regions like enhancers, indicating that they may be disease-promoting by influencing the expression of regulatory risk genes [160]. Epigenetic changes affecting the interaction between the IL-6 gene and the anti-inflammatory gene GPNMB may increase susceptibility for TAK. Specifically, the TAK-associated risk allele rs2069837 A/G in the enhancer region of the IL-6 gene mediates an increased inflammatory response in macrophages through chromatin looping and histone deacetylation that results in suppression of the anti-inflammatory gene GPNMB [145]. Another single nucleotide polymorphism (SNP) found in GCA and TAK that regulates epigenetic gene modification involves the KDM4C gene. KDM4C encodes a trimethylation-specific demethylase that regulates histone methylation and thereby chromatin compaction and transcription [161]. In addition to involvement in cell growth of different cancer cells and cytokine production, KDM4C regulates Jagged1 expression in colonic carcinoma cells [162] and Jagged1 is aberrantly expressed in GCA-affected tissues [114]. However, the impact of the KDM4C SNP and the functional consequences in GCA and TAK remain to be investigated.

### The microbiome as a risk factor for autoimmune vasculitis

Major microbial exposure for humans is their gut microbiome, which may also be subject to age-induced changes. It is now estimated that the human body harbors as many bacterial cells as human cells, emphasizing the potential impact of host-microbial interactions in modulating immunocompetence and disease risk [163, 164]. A variety of factors can shape the human microbiome, including the aging process. Conversely, the microbiome is crucially involved in organismal aging, and gut dysbiosis is recognized in many age-related morbidities [165]. The transfer of the gut microbiome from old mice to young, germ-free mice causes inflammatory processes similar to “inflammaging” [166]. The intestinal microbiome has been implicated in the development of autoimmune diseases such as inflammatory bowel disease (IBD), lupus erythematosus, and multiple sclerosis [167]. Segmental filamentous bacteria (SFB) induce Th17 cells in mice and thereby promote inflammation, whereas Clostridia strains enhance polarization toward anti-inflammatory Treg cells [168, 169]. Aging-induced gut dysbiosis could have a place in GCA, but experimental data supporting a mechanistic link are lacking [170-172].

Likewise, there are limited data about the role of the gut microbiome in TAK. The association of TAK and IBD is intriguing as gut dysbiosis is a key inducer of IBD [173, 174]. Besides changes in diversity and composition of the gut microbiome, alterations in metabolites produced by gut flora, such as short-chain fatty acids (SCFA), have relevance in IBD. Such metabolites essentially participate in various regulatory processes of the host, such as histone modulation, energy metabolism, and immune responses [173,175]. One example is butyrate, which is decreased in IBD and can have immunomodulatory effects by altering the phenotype of macrophages and regulating Treg cell proliferation [176, 177]. These findings and the potential role in TAK warrant further investigation.

The interplay between the gut microbiome, epigenetics, and the immune system highlights the importance of environmental factors such as food, drugs, toxins, and physical activity as modulators of disease risk and disease course. Here, aging-imposed shifts could have a major impact on host-environment interactions, opening the door for lifestyle modifications as promising and cost-effective therapeutic tools [178, 179].

## Conclusions and future perspectives

The primary risk factor for the two granulomatous vasculitides, GCA and TAK, is the age of the patient: the young being susceptible to TAK and the old being susceptible to GCA. The dichotomy of host age in the two diseases should shed light on possible etiologic agents, the composition and function of pathogenic cell populations, and the response patterns of the blood vessel. Aging is an inevitable part of life and is now recognized to cause profound restructuring of the immune system, manifesting with a high risk of older adults to suffer from infections, cancers, neurodegeneration, and cardiovascular disease. The older immune system typically combines immunodeficiency with heightened inflammatory activity, often referred to as “inflammaging” (Fig. 1). Therefore, discussions about the abnormal immunity in GCA need to be placed into the context of immune aging.

The older immune system exemplifies all hallmarks of aging: genomic instability, telomere shortening, altered epigenetic landscape, failing proteostasis, declining mitochondrial fitness, cellular senescence, deficient nutrient sensing, exhaustion of stem cell populations, and accumulation of stem cell mutations. Alignment of these hallmarks of aging with pathogenic events in GCA points toward the age-related failure of CD8^+^ T cells as a disease-relevant mechanism. CD8^+^ T cells, equipped with cytotoxic function, are present in the vasculitic lesions of TAK patients (Fig. 4), but rare in the vasculitic lesions of GCA (Fig. 5). CD8^+^ Treg cells are deficient in GCA patients, where their failure may facilitate the unopposed activity of CD4^+^ effector T cells. Aberrant mTORC1 activation in GCA CD4^+^ T cells mirrors the loss-of-control in the mTOR signaling network of old T cells (Fig. 6). Finally, metabolic signals controlling the PD-1/PD-L1 immune checkpoint, known to lose its tissue-protective function in GCA, may partially weaken due to age-imposed breakdown of intercellular communication.

Emerging data emphasize the impact of the aging process on epigenetic landscapes in immune cells. Increased understanding of how the bioenergetics within and around cells influence cellular function through the generation of ATP and “signaling” metabolites will be informative about cell fate decisions and microenvironmental cues guiding inflammatory processes. Insights from studies of the host microbiome in cancer, cardiovascular disease, and in other autoimmune diseases may provide useful insights into how and why patients form granulomatous infiltrates in arterial walls. Ultimately, the perspective of the patient’s age will not only be important in understanding pathogenesis, but equally impactful in therapeutic management. TAK requires treatment for a lifetime; GCA occurs in individuals with a remaining life expectancy of 1–1.5 decades. Immunosuppressive interventions should be adapted to the youthful immune system of the TAK patients and the compromised immune system of the GCA patient Tables 1 and 2.

## Figures and Tables

**Table 1a:** Adaptations in immune cell function in aging and vasculitis

Cell type	Aging	Giant cell arteritis	Takayasu arteritis
Dendritic cells	↓ Tolerogenicity; ↑ T cell stimulation	↓ Tissue tolerance; ↓ Loss of PD-L1 expression	↑ T cell activation and clonal expansion
Macrophages	↑ Cytokine production; ↑ Inflammasome activation; ↓ Efferocytosis	↑ Cytokine production; ↑ MMP production (basal membrane digestion); ↑ ROS production; ↑ Multinucleation	↑ Pro-inflammatory cytokines; ↑ Multi-nucleated giant cells
CD4^+^ T cells	↓ Naive CD4^+^ pool; ↓ Loss of diversity; ↓ Loss of functional memory cells; ↑ End-differentiated effector memory cells (TEMRA); ↑ Loss of CD28, gain of NK receptors; ↑ Cytotoxicity; ↑ Metabolically reprogrammed	↑ Th1 and Th17 lineage commitment; ↑ Aberrant expression of NK receptor NKG_2_D; ↑ Metabolically reprogrammed ;↑ PD-1^+^ CD4 ^+^ T cells; ↑ NOTCH1 ^+^ CD4 ^+^ T cells; ↑ mTORC1 activation; ↑ CD28 co-stimulation	↑ Predominantly Th1 and Th17
CD8^+^ T cells	↓↓ Naive CD8^+^ pool; ↑↑ Senescence markers; ↑ End-differentiated effector memory cells (TEMRA); ↑ Loss of CD28, gain of NK receptors; ↑ Expression of NK receptors	↓ Minimal numbers in tissue lesions no known pathogenic role	↑ Accumulation in the vascular lesions; ↑ Pathogenic cytotoxicity
Treg	↑ Percentage and absolute number of FOXP3 ^+^ cells; ↓ Inducible Tregs; ↓ Suppressive activity; ↓ Loss of NOX2^+^ CD8^+^ Treg	↓ ↓ Loss of NOX2^+^ CD8 Treg	
B cells	↑ Highly differentiated phenotype; ↓ Antibody response		↑ Anti-endothelial autoantibodies
NK cells	↑ Expanded frequencies and numbers; ↓ Cytotoxic capacity		↑ Part of the inflammatory lesions
Endothelial cells	↑ Inflammatory phenotype; ↑ Adhesion molecules (ICAM, VCAM)	↑ Aberrant Jagged1 on vasa vasorum	↑ mTOR signaling

**Table 1b:** Adaptations in regulatory molecules and pathways in aging and in vasculitis

Molecules and pathways	Aging	GCA	TAK
PD-1/PD-L1 Immune checkpoint	↑ In anti-tumor T cells	↓ PD-L1 low expression on DC and Mϕ; ↓ T cell inhibition	
mTORC1	↑ Signaling in the mTOR network	↑ mTORC1 signaling in CD4 T cells	↑ mTORC1 activity in CD4 T cells; ↑ mTORC1 activity in EC
Sirtuins	↓ Reduced sirtuin activity	↓ SIRT1 in blood mononuclear cells	
AMPK pathway	↓ Reduced AMPK activity; ↓ Reduced mitochondrial activity	↓ Reduced mitochondrial fitness in Mϕ	
NOTCH signaling pathway	↑ Senescence induction by NOTCH signaling	↑ Frequencies of circulating NOTCHl^+^ CD4^+^ and NOTCH4^+^ CD8^+^ T cells; ↑ NOTCH1 signaling ↑ NOTCH4 signaling	
PI3K-Akt pathway	↑ Increased PI3K-AKT signaling upstream of mTOR	↑ increased AKT activation in tissue-residing T cells	
Metalloproteinases	↑ Secretion by senescent cells	↑ MMP-9 and MMP-2 in circulating monocytes; ↑ MMP-9-dependent digestion of basal membrane enabling tissue invasion; ↑ MMP-9 in lesional Mϕ and multinucleated giant cells	
Cytokines	↑ Senescence-associated secretory phenotype (SASP): IL-6, IL-1β, TNF-α, IL-8	↑ IL-6, IFN-γ, TNF-α, IL-17; ↑ VEGF and pentraxin 3	↑ IL-8, CCL2, CCL5, IL-6, IFN-γ, TNF-α, IL-17
Epigenetics	↓ DNA methylation; ↓↑ miRNA expression	↓ Methylation of signature T cell CpG; ↑ Increased miRNAs in lesions	↑ Long-ranged chromatin interactions of disease-associated genetic polymorphisms
Microbiome	↑ Dysbiosis; ↑ Enrichment of pro-inflammatory commensals	↓ Reduced microbial diversity; ↓↑ Altered blood microbiome	↓ Reduced microbial diversity; ↓↑ Altered blood microbiome
